# A Challenging Diagnosis of Gliosarcoma: A Case Report

**DOI:** 10.7759/cureus.85292

**Published:** 2025-06-03

**Authors:** Ioannis P Athinodorou, Kyprianos Kolios, Dimitra Koumoundourou, Constantine Constantoyannis

**Affiliations:** 1 Department of Neurosurgery, University General Hospital of Patras, Patras, GRC; 2 Department of Pathology, University General Hospital of Patras, Patras, GRC

**Keywords:** brain tumor, central nervous system, glioblastoma, gliosarcoma, neurosurgery

## Abstract

Gliosarcoma (GS) is a rare variant of glioblastoma (GBM) that usually affects middle-aged adults. The radiological characteristics of GS are similar to the radiological features observed in GBM. We present the case of a 74-year-old male patient who presented with neurological symptoms associated with a brain tumor. The patient had a medical history of prostate cancer, and MRI findings suggested the diagnosis of brain metastasis or GBM. However, postoperative histopathological examination of the tissue specimen confirmed a diagnosis of GS. GS can mimic several entities and should be included in the differential diagnosis of metastasis-like brain masses.

## Introduction

Gliosarcoma (GS) is a rare aggressive tumor of the central nervous system consisting of glial and mesenchymal components, usually affecting middle-aged men. It is a subtype of glioblastoma (GBM) according to the WHO Health Organization 2021 classification and has a poor prognosis [1-10]. The radiological and clinical features of GS can be similar to those of GBM [11]. We present a case of multilobar GS in a 74-year-old male patient with a history of prostate cancer, which was initially through the patient’s history and radiological characteristics thought to be a central nervous system metastasis. This case of pre-operative misdiagnosis highlights the importance of pathology to achieve the correct diagnosis and the necessity to include a wide spectrum of entities in the differential diagnosis of a central nervous system mass.

## Case presentation

**Figure 1:**
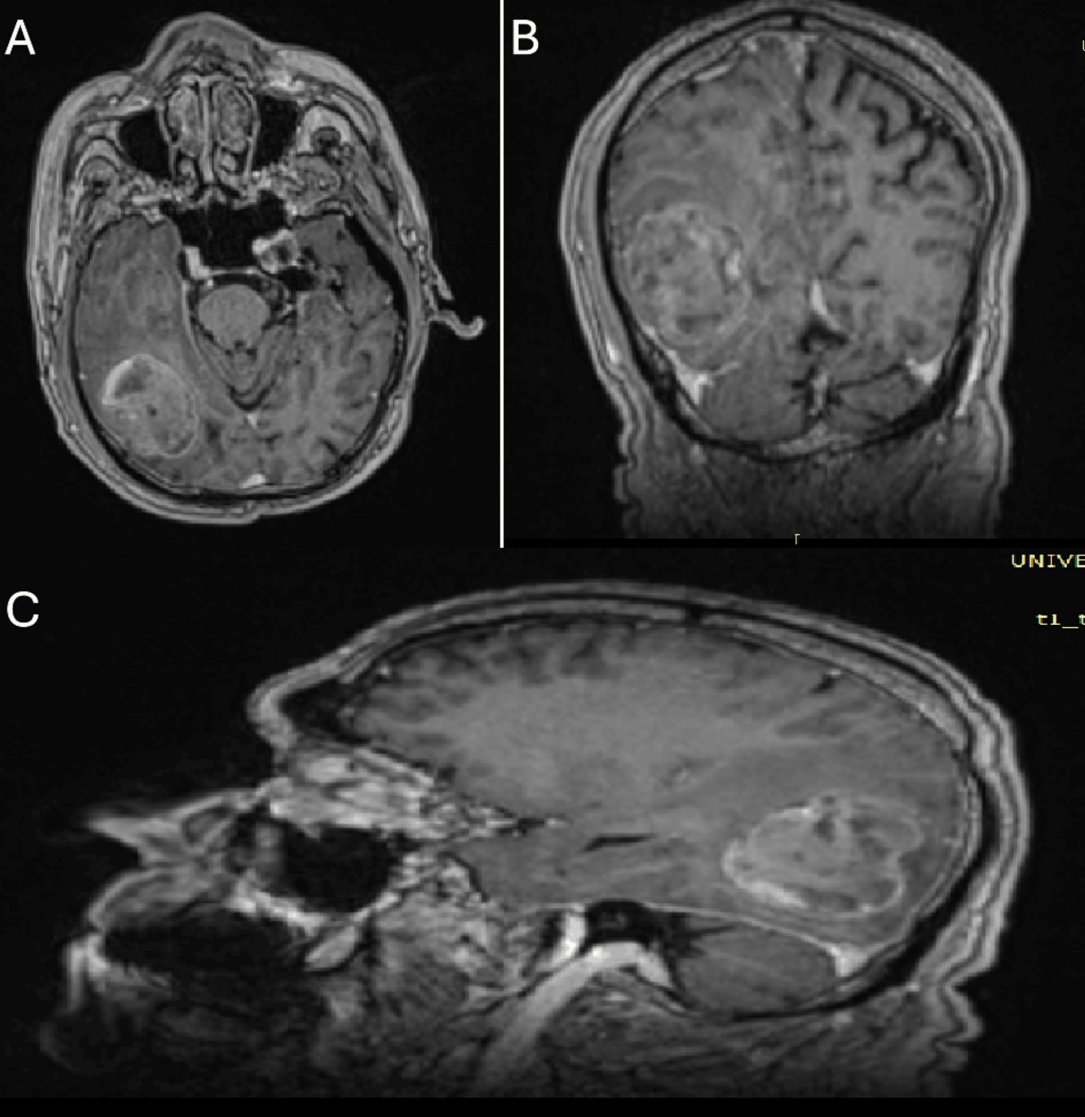
Pre-operative MRI T1-weighted image + Gd The pre-operative magnetic resonance imaging (MRI) study, performed with a gadolinium-based contrast agent, demonstrated a lesion exhibiting peripheral enhancement. The upper left image corresponds to an axial view (A), the upper right image represents a coronal view (B), and the lower image depicts a sagittal view (C).

**Figure 2:**
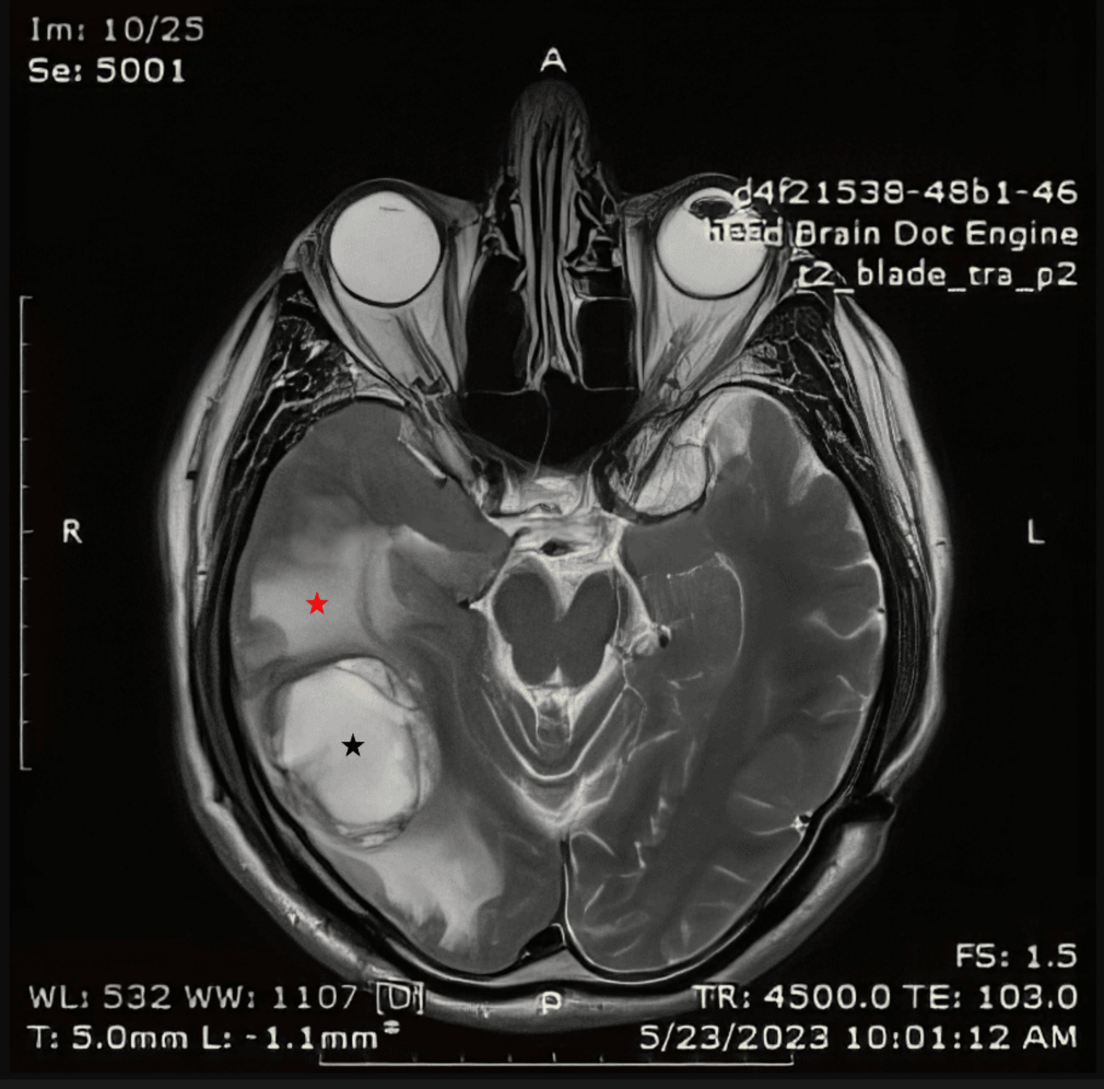
T2-weighted image The MRI image shows a hyperintense mass originating in the right temporal lobe (black star) with edema of the surrounding tissue (red star)

A 74-year-old male patient presented with gait disturbance and mild paresis of the left upper extremity starting from the month prior to presentation. Brain magnetic resonance imaging (MRI) revealed a well-demarcated parietal-temporal-occipital mass with metastasis-like morphology. The dimensions of the mass were approximately 3.5cm x 4.0cm x 4.5cm. The mass was iso- to hypointense on T1 and hyperintense on T2. Peripheral enhancement on T1-gd+ images and edema surrounding the mass were also observed (Figure 1 and Figure 2).

**Figure 3:**
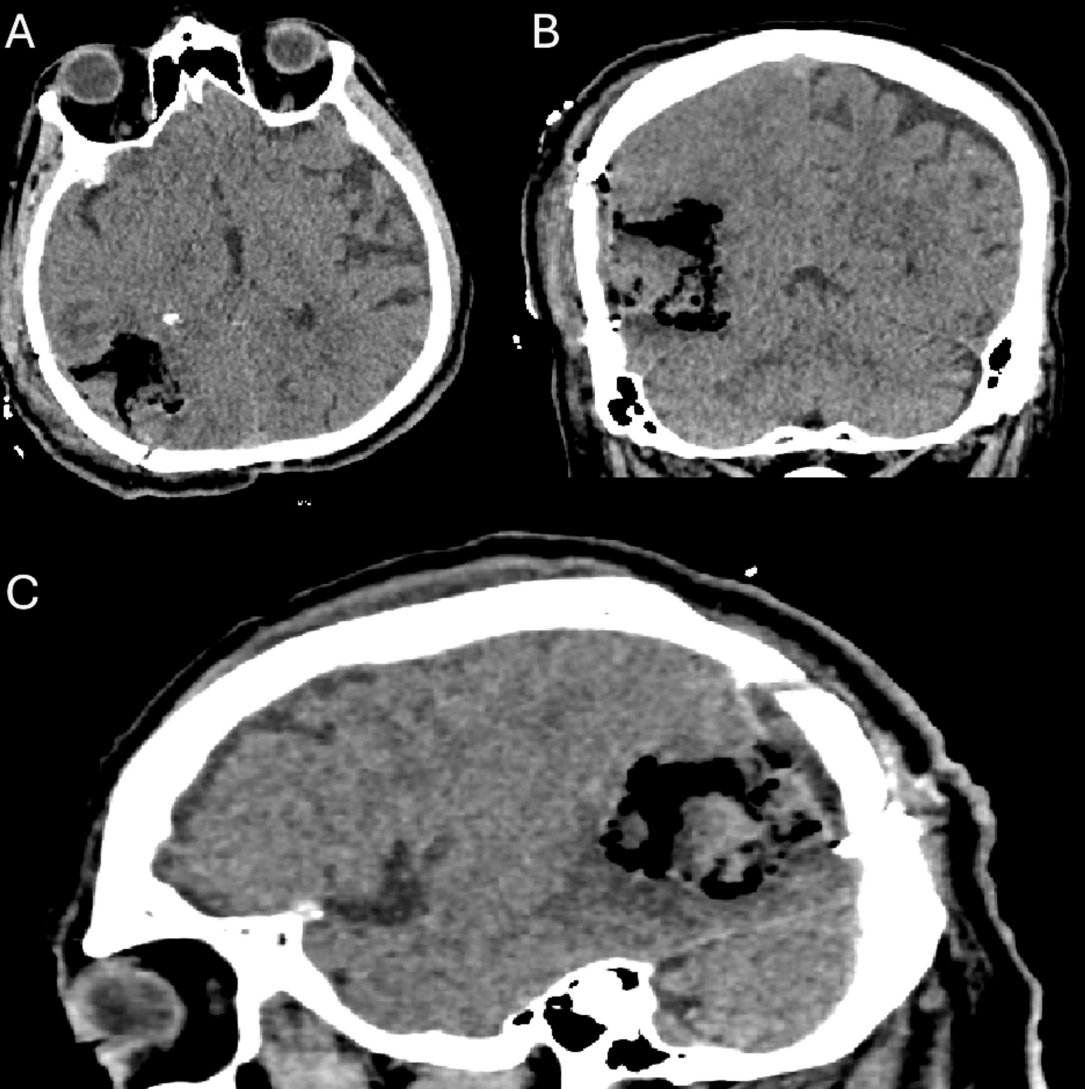
Post-operative CT scan The post-operative CT scan demonstrates the tumor cavity in three planes: axial (A), coronal (B), and sagittal (C).

The patient had a history of prostate cancer that was treated with surgery and radiotherapy; hence, the mass was initially thought to be a metastasis. Other comorbidities included atrial fibrillation, dyslipidemia, and arterial hypertension. Surgery was scheduled one week later. A temporoparietal craniotomy was performed with the help of MRI navigation. The tumor had a small dural attachment and was composed mainly of solid regions. The macroscopically affected part of the dura was removed. Tumor excision was performed using ultrasonic aspiration and bipolar coagulation. A gross total resection was achieved. The tissue specimens were sent for histopathological examination. Postoperatively, the patient was clinically and neurologically stable with a GCS score of 15, and no complications were observed; therefore, he was discharged on the sixth postoperative day. Postoperative computed tomography (CT) revealed a tumor cavity (Figure 3).

**Figure 4:**
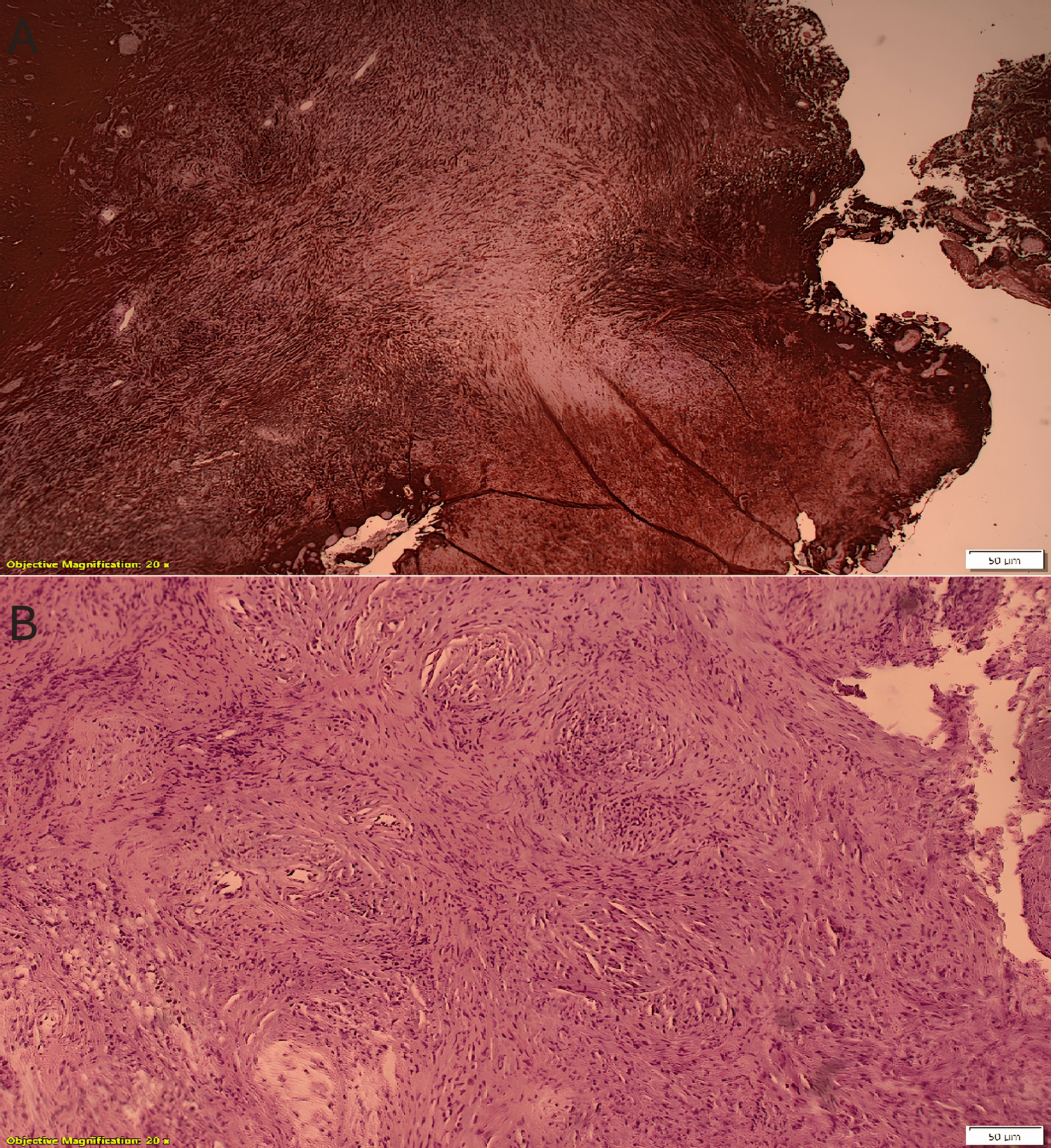
Tissue specimen A. GFAP+ cells indicating the glial component; Β. Cells with spindle morphology indicating the sarcomatous component

Despite the clinical and radiological diagnosis of metastasis, histopathological examination revealed GBM (WHO grade IV) with the characteristic of GS. Microscopically, the tumor consisted of pleomorphic astrocytes with nuclear atypia, several mitoses, and palisading necrosis foci. The Ki-67 index was 12% which supports a moderate proliferative activity. Microvascular proliferation was also observed. Some tumor areas showed a sarcomatous component with atypical spindle cells mimicking a sarcoma. Immunohistochemically, the neoplastic cells expressed GFAP (even in sarcomatous areas, which were also positive for actin and vimentin) (Figure 4).

Immunohistochemical expression of cytokeratins (ΑΕ3, CK8/18) and PSA was negative. Due to the histopathological diagnosis, the patient had received additional radiotherapy, but unfortunately the progress of the disease was rapid and as a result of the patient’s poor performance clinical status, temozolomide was not administered. The patient died a month later. 

## Discussion

GS is a subtype of GBM that accounts for approximately 2% of them and 2-8% of high-grade gliomas [1-5]. More specifically, it is classified as a WHO grade IV variant of GBM [6-8]. It is usually present during the 5th to 7th decade of life with a male predominance [2,4,6,9,12,13]. In addition, predominance has been observed in white non-Hispanic races [6]. GS can be presented with a variety of signs and symptoms including headaches, seizures, hemiplegia and hemiparesis, loss of consciousness, cognitive changes, and speech disturbances [1,2,3,6]. However, as Peckham et al. stated in their article, a notable number of patients can also be asymptomatic [1]. GS can be considered primary if they appear de novo without any history of GBM. However, they can also be deemed as secondary in cases where the patient has a history of GBM, which has received treatment with resection and radiation. Nevertheless, in the absence of GBM in patients who received intracranial radiation, GS is classified as radiation-induced [4,14]. The vast majority of GS are supratentorial, with the temporal lobe being the most common site of the tumor; however, the frontal lobe is sometimes noted as the most common site. The parietal and occipital lobes are rare [2,4,12,14,15]. Frandsen et al. stated in their research that the multilobar location was the second most common location following the temporal lobe [4].

The 2021 fifth edition of WHO Classification of the Central Nervous System Tumors is an update of the 2016 edition which was based on molecular and histological parameters. GS is classified in the category of diffuse astrocytic and oligodendroglial tumors and the subcategory of GBM, IDH-wildtype along with giant cell glioblastoma and epithelioid glioblastoma [10]. Two theories have been proposed for the origin of GBM. First, the sarcomatous component originates from the neoplastic transformation of hyperplastic vessels of the glioma. The second assumption is that mesenchymatous differentiation of a glioma occurs, which is supportive of a monoclonal origin of GS [3,12,15].

Histologically, they have a dual cell population, which creates mesenchymal and glial components. Hypercellularity, necrosis, and high mitotic activity were observed in both populations. The glial component was characterized by astrocytic morphology, while spindle cells were observed in the sarcomatous [1,6,8,12]. The mesenchymal part is commonly a fibrosarcoma; however, osteosarcoma, chondrosarcoma, angiosarcoma, and rhabdomyosarcoma can be observed as well [16]. Immunohistochemistry can also help differentiate between biphasic cell populations. Glial cells are GFAP (+), while the mesenchymal cells are Reticulin (+) [6,12]. Salvati et al. reported that there are two subtypes of GS, a gliomatous predominant and a sarcomatous predominant, which are similar to meningioma in radiological and surgical characteristics and shows a better overall survival [9].

The radiological images of GS present similarities and overlap with the radiological images of GBMs [1,2]. At the same time, the biphasic histological characteristics of GS present with distinct MRI findings. Enhancement has been observed in the vast majority of GS [1,15,17]. The meningioma-like appearance that represents a homogeneously diffused enhanced mass with a well-bound rim enhancement, while, on the other hand the glial-derived tumors often present without total rim enhancement and with a heterogeneously enhancing mass [2,6]. However, this formulation is not a rule, with images often overlapping, presenting a mixed pattern, and showing additional features, such as necrotic and cystic masses [1,17,18,19].

As Chourmouzi et al. stated in their article, GS and dural metastasis should be included in the differential diagnosis of meningioma and vice versa. Therefore, a dural lesion can represent any of these entities [20]. Conclusively, in the differential diagnosis of GS, metastasis, abscesses, PNETs, meningiomas, astroblastomas, and GBMs [17,19,20]. Han et al. reported in their case series of 15 people that in two cases, the initial diagnosis was metastasis, while Zhang et al. reported the same in three out of 54 patients [17,19]. These cases of preoperative misdiagnosis agree with our case.

The gross total resection, postsurgical radiotherapy, and chemotherapy with temozolomide are currently the standard treatment guideline for GS, which are comparable to the therapeutic approach for GBMs [1,2,5,7,18]. Recent publications, in contrast to previous studies, suggest that the use of TMZ-based chemotherapy results in favorable outcomes and extended survival. Especially in patients with MGMT promoter methylation, temozolomide is more beneficial, with improved survival [5,18]. Compulsory implementation of radiotherapy has been proposed to enhance the extended results in patients, as it potentially leads to increased survival by 8-15 weeks in the long run. Gross total resection seems to be the most decisive factor for overall survival [5]. Bevacizumab shows promising results as an approach, but the limited data available hampers definitive conclusions about its effectiveness [18].

In contrast to GBMs, GS tends to metastasize more frequently. The documented literature rate of extracranial metastatic lesions is approximately 11% [4,5,18]. The metastatic capability of GS is attributed to its sarcomatous component, signifying the inherent tendency of sarcomatous neoplasms to spread via the bloodstream. Indeed, the histopathological analysis of metastatic tumors revealed a predominant sarcomatous composition, which reinforces the notion that the sarcomatous component exhibits a heightened propensity for hematogenous spread in relation to the glial component. GS metastases are mostly observed in the lungs, liver, and lymph nodes [3,7,18].

As a WHO grade IV tumor, patients with GS have poor overall survival. The median survival was estimated at 13.9 months, while in untreated patients, it was four months. There are some main predictive factors for overall survival, such as the extent of resection, use of adjuvant therapies, and age [5,18]. In patients who underwent gross total resection, radiotherapy, and chemotherapy with temozolomide, survival improved and extended. The extent of resection performed was more likely to be complete in the meningioma-like subtype [2,3]. Thus, the sarcomatous subtype exhibits a more favorable prognosis with higher median survival rates due to complete resection [9,18].

## Conclusions

To conclude, we reported a case of supratentorial multilobar GS in a patient with characteristics that corresponded to those reported in the literature. However, the initial diagnosis, through radiology tests and the patient’s previous medical history, was different from the pathology report. Thus, although GS is an infrequent entity, it should be added to the differential diagnosis list when a patient with a specific profile presents with a GBM, metastasis, or meningioma-like mass. A high index of clinical suspicion in patients with the appropriate profile alongside with immunohistochemistry are fundamental for diagnostic accuracy. Further studies, prospective and metanalyses, are essential to enhance our understanding of this entity.
